# Homebrew Photolithography for the Rapid and Low-Cost, “Do It Yourself” Prototyping of Microfluidic Devices

**DOI:** 10.1021/acsomega.3c05544

**Published:** 2023-09-15

**Authors:** Daniel Todd, Natalio Krasnogor

**Affiliations:** Interdisciplinary Computing and Complex BioSystems, ICOS, Newcastle University, Newcastle upon Tyne NE4 5TG, U.K.

## Abstract

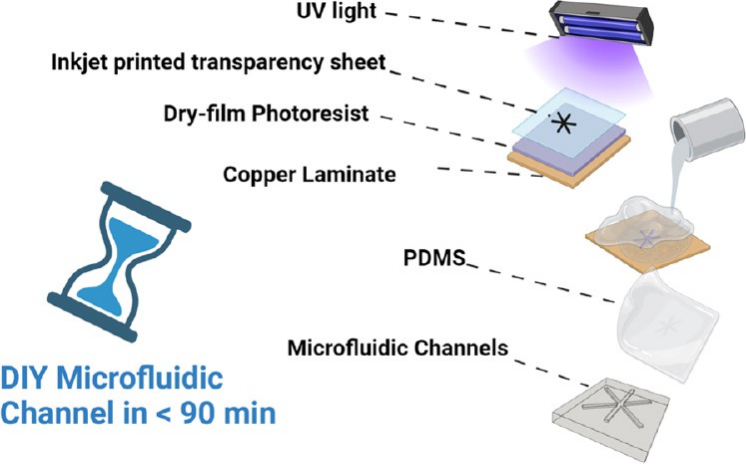

Photolithography is the foundational process at the root of micro-electromechanical (MEMS) and microfluidic systems manufacture. The process is descendant from the semiconductor industry, originating from printed circuit board and microprocessor fabrication, itself historically performed in a cleanroom environment utilizing expensive, specialist microfabrication equipment. Consequently, these conditions prove cost-prohibitive and pose a large barrier to entry. We present a novel homebrew, “do-it-yourself” method for performing photolithography to produce master mold wafers using only household appliances and homemade equipment at the bench side, outside of a cleanroom, producing a range of designs including spiral, serpentine, rectangular, and circulatory. Our homebrew processes result in the production of microfluidic channels with feature resolution of ∼85 μm width and 50 μm height utilizing inkjet-printed photomasks on transparency film to expose dry-film photoresist. From start to finish, the entire process takes under <90 min and costs <£300. With SU8 epoxy negative photoresist and a chrome photomask, our low-cost UV exposure apparatus and homemade spincoater could be used to produce PDMS devices containing large arrays of identical microwells measuring 4.4 μm in diameter. We show that our homebrew method produces both rectangular and spiral microfluidic channels with better results than can be achieved by SLA 3D printing by comparison, and amenable to bonding into multilayer functional microfluidic devices. As these methods are fundamental to microfluidics manufacture, we envision that this work will be of value to researchers across a broad range of disciplines, such as those working in resource-constrained countries or conditions, with many and widely varying applications.

## 1 Introduction

Microfluidics as a technology has widespread application potential; however, reality has fallen short with respect to original predictions of ubiquity. Part of this could be due to the considerable barriers to entry, namely, access to expensive, specialist microfabrication machinery and a cleanroom environment. Thus, the application of microfluidics is unfortunately restricted to the few appropriately resourced laboratories. While commercial microfluidic wafer production offers convenience and high-quality results, there are compelling reasons why do-it-yourself (DIY) photolithography can be a better choice for microfluidic device prototyping, chiefly, Cost-effectiveness and rapid prototype design iteration. DIY photolithography offers a cost-effective alternative to commercial microfluidic wafer production; the equipment and materials required for photolithography are very affordable and can be reused for multiple fabrication runs. By eliminating the expenses associated with outsourcing production, researchers and small laboratories can significantly reduce costs and allocate resources more efficiently. Second, a DIY approach provides the capacity for rapid prototyping and iterative design. Researchers can quickly modify their designs, fabricate new microfluidic devices, and test them without relying on external service providers. This iterative process allows for faster design optimization and accelerates the development cycle, facilitating innovation and exploration of novel microfluidic concepts. This DIY rapid-prototyping capacity offers much design flexibility and potential for customization: With DIY photolithography, researchers have complete control over the design and customization of their microfluidic devices. Commercial services often have limitations on design flexibility and customization options, whereas DIY photolithography empowers researchers to tailor their designs to specific experimental needs, incorporate unique features, and experiment with different layouts and dimensions. By having direct access to the fabrication process, such as channel dimensions, surface chemistries, and material selection, researchers wield a greater degree of control, which is crucial for tailoring microfluidic devices to specific applications, optimizing performance, and achieving desired functionalities. Researchers can experiment with different fabrication parameters to improve device characteristics and adapt them to specific experimental requirements.

It is important to note that DIY photolithography may unavoidably require an initial (albeit at a significantly lower value than for traditional routes) time/cost investment in equipment, training, and expertise. However, the long-term cost savings, design flexibility, rapid prototyping capabilities, and experimental freedom outweigh these considerations for many researchers. DIY photolithography empowers researchers to take control of their microfluidic device fabrication process, unleashing their creativity and driving innovation in the field of microfluidics. To this end, we introduce our Homebrew approach to photolithography to combat this initial barrier to onboarding and lower the learning curve.

### 1.1 The Process

**Figure 1:**
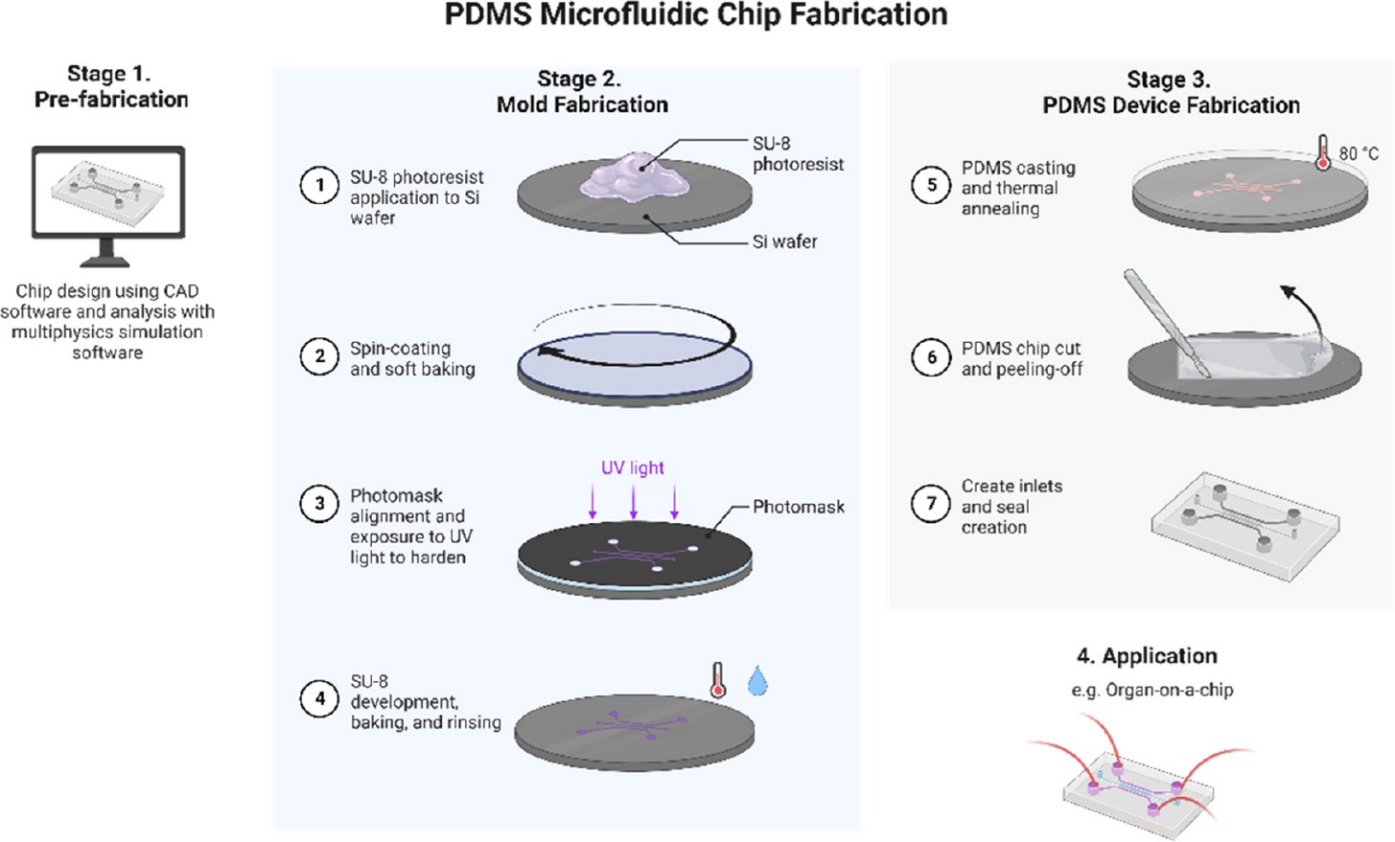
Overall typical workflow for microfluidic chip production from CAD design to assembled device.

The first step in the traditional microfluidics production workflow is the production of the design using CAD software (stage 1 in Figure 1). The bottleneck to wider microfluidics adoption occurs at stage 2 of the process flowchart, with the production of a wafer template via photolithography, which is then used to mold microfluidic devices, most commonly in Poly(dimethylsiloxane) (PDMS). Photolithography involves the fabrication of a master on a silicon wafer. This is achieved through the coating of a light-sensitive polymer (steps 1 and 2 of stage 2), followed by light exposure through a patterned photomask (step 3), and subsequent baking and chemical development to cause selective dissolution (step 4) leaving patterned features to be used as the template for PDMS soft lithography. This output is the template for the production of microfluidic devices.^1,2^ To produce a microfluidic chip, a photoresist patterned wafer is required, which itself requires a photomask possessing opaque and translucent features.^3^ The photomask is key to achieving a selective dissolution of photosensitive polymer, and different materials are used to produce photomasks including soda lime, quartz, and films such as polyester.^4^ Conventionally, a photomask is obtained through designing (or having a company design one for you) a pattern and then outsourcing the production of a photomask to an external company that charges for the production of the wafer—depending on pattern complexity, this can be both an expensive and lengthy process. The output of this process is the production of a single design, often on a single relatively fragile silicon wafer. Any damage or changes to designs would require having to pay again and repeat the process for every minute modification to the design, which is incompatible with the design, build, and test engineering principle of exploratory development and negates any ability to rapidly prototype. In light of this constraint, various alternative methods have been investigated, including the use of 35 mm photographic negatives as photomasks,^5^ and even maskless photolithography,^6^ including the use of standard inkjet printers both to produce a photomask by printing negative designs onto transparency film^7^ and more direct methods of inkjet print-patterning onto surfaces using a variety of inks. Inkjet printers can produce linewidths down to 50 μm.^8^

**Figure 2:**
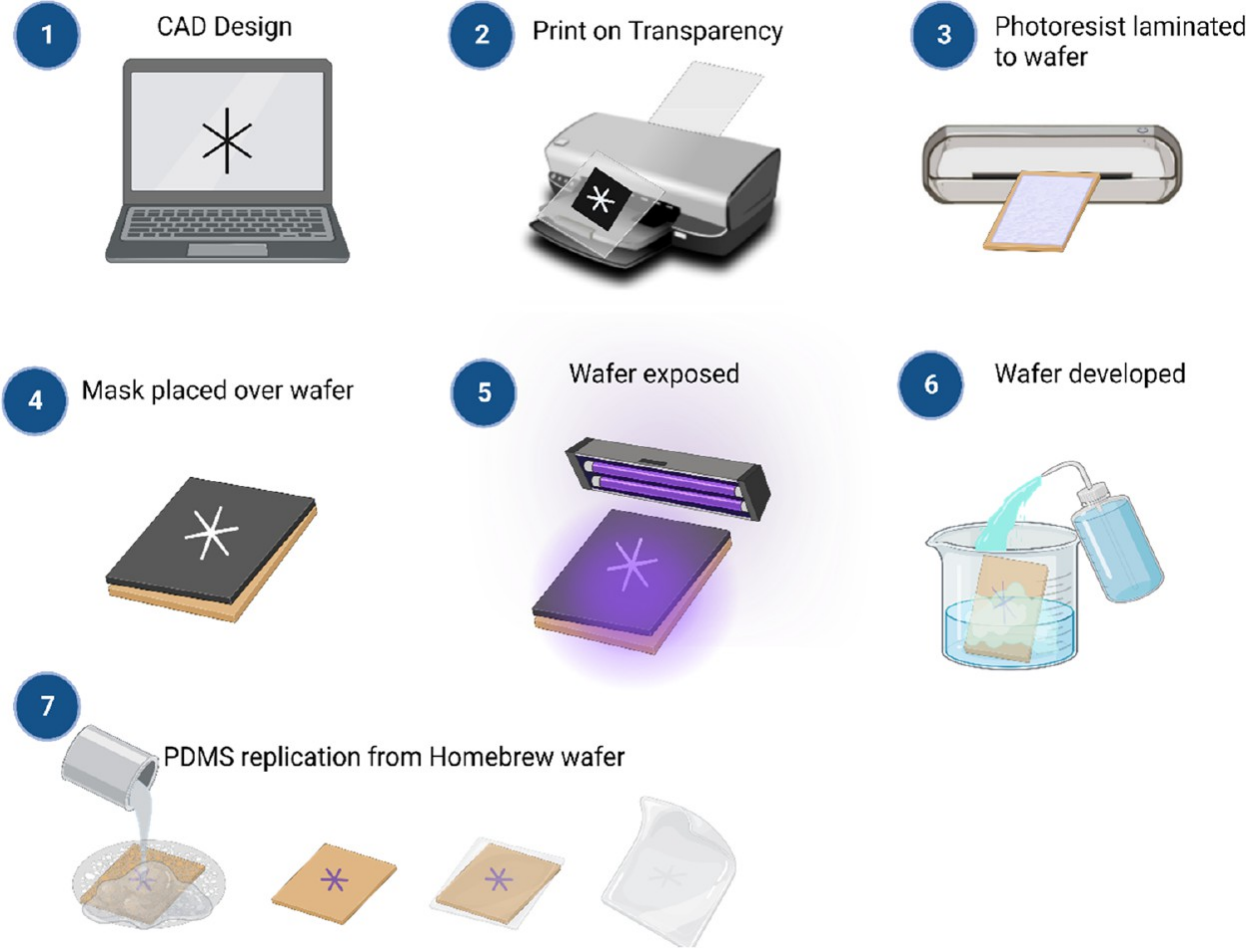
Schematic process steps of homebrew photolithography. (**1**) CAD design of microfluidic device features, (**2**) printing on transparency film to function as homebrew photomask, (**3**) lamination of dry-film photoresist on epoxy-clad copper laminate, (**4-5**) UV exposure with homebrew photomask fixed in place for selective hardening of desired shapes, development in sodium carbonate to wash away unexposed areas, (**6**) rinsing clean, and (7) finally PDMS replication from homebrew wafer.

Another impediment to the rapid prototyping of microfluidic devices through conventional means is the high cost of SU8 photoresist^9^ (typically reaching ∼£1000 each, depending on the desired depth of layer after spin coating.) Another caveat to the use of SU8 is the uneven flatness even following spin coating, which is characterized by a so-called “edge bead” at the outer edges, which if not removed correctly can prevent the photomask sitting flat atop the photoresist, resulting in reduced resolution of the resulting pattern. Such a high cost also warrants the search for alternatives, and indeed various dry-film resists (DFRs), and photosensitive polymer films were developed for the fabrication of printed circuit boards that have the benefit of good-flatness/no edge-bead occurrence; they are also simpler/more straightforward to manipulate and apply.^10^ In this work, we demonstrate the production of a range of microfluidic master molds of various shapes and dimensions using inkjet-printed photomasks on acetate sheets from a standard home and office printer in combination with photosensitive DFR film applied to copper clad epoxy tiles using a typical laminator appliance. We expose the photoresist through the homemade photomasks by using a UV LED black-light floodlight, much like those used in aquariums, rave parties, or used to cure resins. A schematic overview of our homebrew method is shown in Figure 2. The method has obvious advantages over older methods which utilized elaborate and complex processes involving increasingly harsh and hazardous chemicals such as hydrochloric acid and chloroform.^11^ Our process is also quick, allowing us to go from zero to a completed PDMS chip in 90 minutes, again, clearly advantageous over other methods that involve significant time-sinks associated with the fabrication protocols.^12,13^Table 1 highlights some of these techniques and their comparative feature resolutions, speed, and cost considerations.

**Table 1:** Comparison of Different Methods of Microfluidic Master Mold Production

microfabrication technology	minimum feature size	lead time	overheads	ref:
X-ray lithography (LIGA)	0.1–3 μm	2 weeks	∼$ 60K	(14)
laser ablation	1 μm	2 weeks	>£100K	(15)
wet-etch	3 μm	1–2 weeks	£50K	(16)
dry-etch	100 nm	2–4 weeks	£20K	(17)
deep reactive ion etching	0.2 μm	2–4 weeks	∼£50K	(18)
e-beam lithography	<8 nm	2–4 weeks	∼$100K	(19)
wax printing	275 μm	<1 h	$2000	(20)
SLA 3D printing	100–500 μm	av. 1–2 h	£500	(21)
homebrew method*	85–100 μm	90 min total	£275	this work*

However, for these existing methods, a trade-off exists between feature resolution achievable, complexity, and completion time. To this end, we set out to develop a simple, low-cost, rapid prototyping method for the production of functional microfluidic channels to provide researchers with greater control and flexibility in their microfluidic experiments.

## 2 Experimental Section

### 2.1 Materials and Methods

#### 2.1.1 Photomask CAD Design

Several transparency photomasks (spiral, serpentine, rectangular straight channels, circulatory) were hand-drawn with CAD software (Sketchup Pro).

#### 2.1.2 Photomask Printing

Photomasks were printed onto an OHP transparency film (Amazon) with either a desktop inkjet printer (HP DeskJet 3630, 4800 × 1200 dpi) or a toner-based laser printer (Bizhub C368, 1200 × 1200 dpi) using standard, readily available inks complementary to the printer recommended as standard by the manufacturer (Amazon).

#### 2.1.3 Wafer Production

Master mold wafers were either produced through one of two means:

(a) spin-coating SU8 (Agas Chemicals) using a homemade spincoater (see the Supporting Information) on conventional 4-inch silicon wafers (Darwin Microfluidics) for 30 s at 1000 rpm to achieve a deposited layer of 4 μm thickness. The resulting edge-bead at the wafer edge was then removed manually via an acetone-soaked swab, which was swept across the outer border, removing the edge bead. The wafer was then soft-baked for 25 min and left to cool. Once cool, the transparency photomask was manually placed onto the wafer and held in place with bulldog clips, and off-target areas were covered in dark tape to prevent unwanted exposure. The wafer was then exposed to UV light through an LED floodlight emitting light at ∼2 mW per cm^2^ for a duration of 40 s. Following exposure, the wafer was then post-exposure baked for a further 12 min and left to cool, and then developed for 2 min in SU8 developer and rinsed clean with isopropyl alcohol.
(b) Portable PCB Photosensitive Dry Film (30 cm × 5 m) Photoresist Sheeting was purchased from Amazon and applied to Double-Sided Copper Clad Laminate DIY Prototyping PCB Board using a standard Crenova A4 Laminator appliance (also purchased from Amazon.) Following lamination, the surface of the board was passed over with a heat gun on a low setting with care taken to avoid the nozzle getting too close and overheating the surface. The outer protective layer was then carefully removed. The transparency photomask was then placed manually onto the board beneath a glass slide to ensure flatness and clamped in place using bulldog clips. Off-target exposed areas were covered using black electrical tape. The wafer was then exposed to UV light through an LED floodlight emitting light at ∼2 mW per cm^2^ for a duration of 1 min 40 s. After exposure, the slide and photomask were removed, and the surface was briefly passed over with a heat gun on a low setting again. The board was then developed through immersion and gradual agitation in a solution of sodium carbonate, purchased from Amazon (24 g per liter of water), and rinsed in a solution of warm water.

#### 2.1.4 Microfluidic Chip Fabrication

Channels containing microfluidic chips were fabricated by standard soft lithography process using PDMS (poly(dimethylsiloxane), Sylgard 184, Dow Corning). Briefly, all molds were silanized under vacuum using trichloro (1H,1H,2H,2H-perfluorooctyl)silane (PFOCTS) (Sigma-Aldrich U.K.). PDMS was mixed with a curing agent at a ratio of 10:1, degassed for 30 min and then poured onto the masters, a silicon wafer, an SLA 3D-printed mold, or a copper clad laminate wafer. The wafers were then placed on a hot plate at 80° for 2 h to cure. PDMS replicas were peeled from the masters, cut to size, and placed on a microscope slide for inspection under a microscope. 3 μm polystyrene fluorescent (FLASH-red) microspheres were purchased from Bangs Laboratories.

#### 2.1.5 Imaging

Overview pictures were collected using a Huawei P30 smartphone camera. PDMS replicas and photomasks close-up were imaged using a Nikon Ti2 inverted microscope in either bright-field or epifluorescence modes.

### 2.2 Analysis

Measurements of widths, lengths, and intensity profiles were made using Nikon NIS Elements software.

### 2.3 3D Printing

The same hand-drawn 3D CAD files of the rectangular and spiral channels were rendered in three dimensions at a 400 μm positive feature height (the minimum the printer can reproduce with high-fidelity from prior testing). The standard recommended slicer software Chitubox (v1.8.1 software) recommends a 500 μm layer height by default. The initial .skp SketchUp file was exported as an STL file and imported into Chitubox where the software converted the file into a .ctb in preparation for printing. The manufacturer states a minimum feature resolution theoretically achievable of 50 μm, though we found from previous experimentation that this was not practically achievable as a series of complex trade-offs rendered this unobtainable. For instance, smaller feature sizes are producible from reductions in layer thickness, in order to ensure that the correct shapes are fully formed, longer exposure times are required, a consequence of this being that during this exposure, any trapped liquid resin in any spaces while become inevitably polymerized during this exposure, often resulting in undesirable structural perturbations. We note that these limitations are in part inherent with the choice of 3D printer employed, which was the Elegoo Mars2 Pro. With customized or custom-produced 3D printers, some of these limitations could be at least partly alleviated. The resin employed for the production of the molds was ELEGOO LCD UV 405 nm ABS-Like 3D Printer Resin (Elegoo). Exposure times were 7 s for the bottom layers and 3 s for normal layers. The layer thickness was set at 400 μm.

All molds were rinsed with isopropyl alcohol and washed and cured using the Elegoo Mercury plus 2 in 1 wash and curing station (Elegoo) for the recommended range of 10–20 min for cure and wash, respectively. The pieces were left to air-dry. Once the molds had been cleaned and cured, we placed the molds in an oven at 90° for 18 h to ensure curing was complete, in line with several reports from other groups to ensure complete curing to combat potential curing inhibition at the subsequent replica molding stage.

## 3 Results and Discussion

To evaluate our homebrew method, several test microfluidic devices were fabricated.

The central focus was on two designs: a spiral channel and a regular rectangular channel series. These designs were fabricated into chips containing channel widths ranging from ∼650 to 85 μm for the straight channels in order to determine the range of viable devices. Viability was assessed by bonding the channels atop a planar array of microwells and using the homebrew channel to introduce in separate trials solutions of 3 μm fluorescent polystyrene microspheres and Fluorescein with mineral oil in order to evaluate the ability of the channel to carry the solutions and assess the degree of dispersal/seeding and sealing that is achievable across planar arrays.

**Figure 3:**
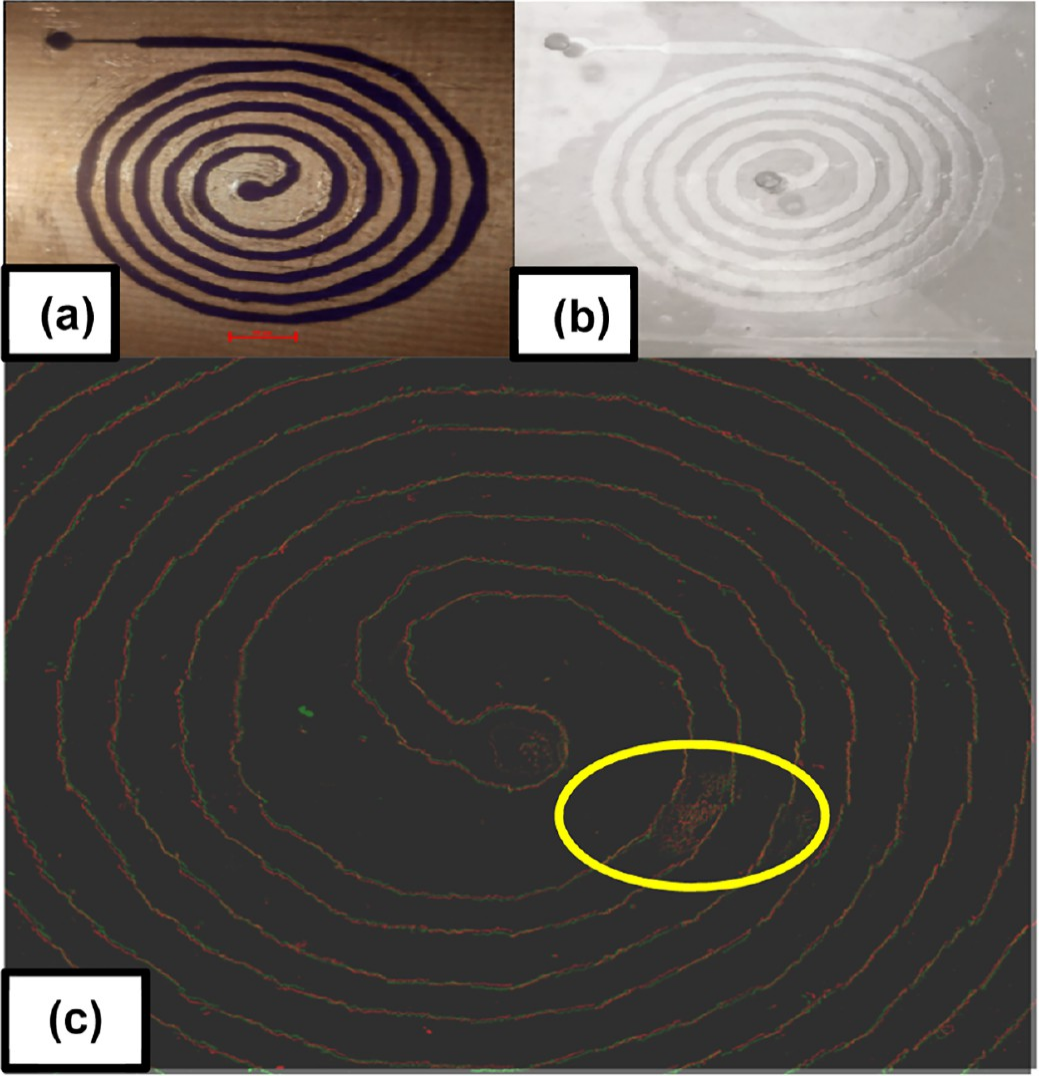
(a) Homebrew wafer, (b) replica in PDMS, and (c) outline of two replicas overlapped, demonstrating effective reproducibility of nonstandard wall geometry, showing the corrugated walls are easily reproducible. Outlines have been positioned close side by side rather than directly on top to ensure each is equally visible and the matching patterns are most clear. While channels are largely clear, in this example, any roughness or unevenness of the dry-film photoresist is faithfully also copied onto resulting replicas, as can be seen here in the first turn of the channel (circled in yellow). As such, it is important to ensure that dry-film adheres flat to the copper wafer. Scale bar equal to 2 mm.

The biggest contributor to the resulting resolution achievable was the choice of photoresist employed, with the more expensive SU8 capable of producing smaller features than the cheap dry film. With the dry film, the smallest features of channels that could be reliably reproduced were of ∼85 to 100 μm, a restriction imposed largely by the type of photosensitive polymer used, which was a ubiquitous, general purpose, cheap (<£20 each) widely available film from Amazon for DIY PCB production. With other (albeit more expensive) photopolymers such as ADEX film, smaller features should be achievable. Overall, our homebrew process could be achieved entirely at a low cost, <£300, as shown in Table 2, which is significantly cheaper than the alternative methods listed in Table 1, in addition to being significantly faster from start to finish (<90 min). Our homebrew process involves the production of a copper wafer (Figure 3a), which can be utilized in replica molding to produce faithful, reproducible microfluidic channels (Figure 3b).

**Table 2:** Cost Breakdown of Homebrew Photolithography Method. See the SI for Breakdown of Homemade Spincoater and Instructions for Assembly

item	cost	unit amount
transparency film (inkjet)	£9.50	20 sheets
transparency film (toner)	£12.00	50 sheets
copper clad epoxy laminate	£7.00	10 pcs
laminator	£19.99	1
handheld hot air gun	£10.69	1
bulldog clips	£2.19	1 pack
black electrical tape	£1.50	1 roll
UV LED Light	£48.99	1
sodium carbonate	£5.49	500 g
photoresist film	£8.16	5 M
inkjet printer	£34.99	HP Deskjet 2720e all-in-one
inkjet printer inks	£13.07	HP 305 3YM6OAE Tricolor
homebrew spincoater*	£100	
**Total:**	**£273.57**	

**Figure 4:**
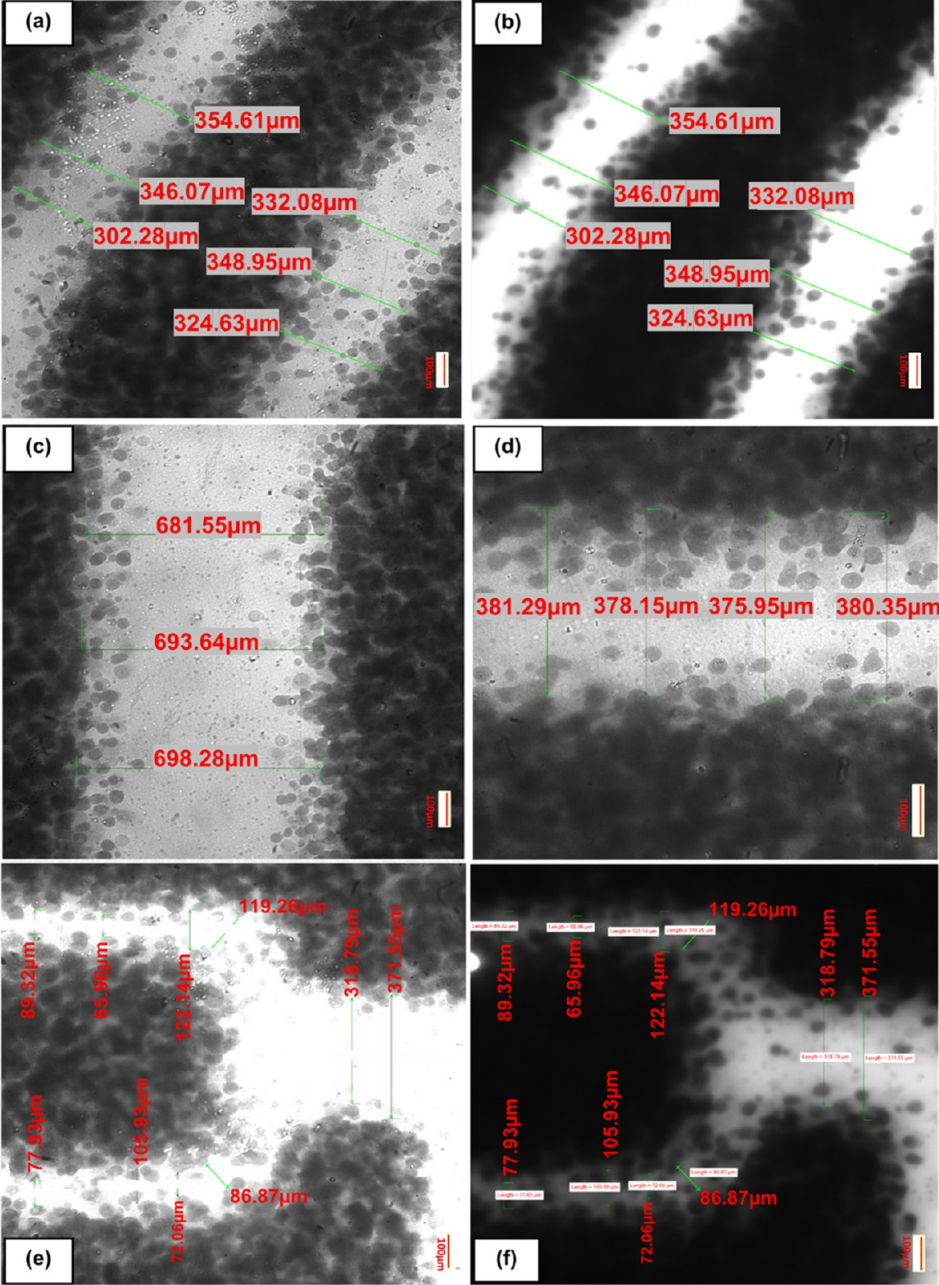
Microscope images of homebrew photomasks inkjet-printed on transparency films. Scale bars are 100 μm. (a, e) Bright-field and (b, f) 365 nm wavelength light show that both the light wavelength and intensity can affect the resulting homebrew channel production. (c, d) As the space (resolution) between two features decreases, the channel uniformity is reduced as more ink blots from the transparency mask overrun into the main channel features, demonstrating that channel feature reduction is controlled at least partially by the minimum print resolution achievable from the printer used: (a), (b) 354.61, 346.07, 302.28, 332.06, 348.95, 324.63 μm, (c) 681.55, 693.64, 698.28 μm, (d) 381.29, 378.15, 375.95, 380.35 μm; (e, f) 89.32, 65.96, 122.14, 119.26, 318.79, 371.55, 86.87, 72.05, 72.06, 105.99,77.93 μm.

Figure 3c shows two composite images of PDMS replicas possessing a spiral channel of size (50 μm height and 300 μm channel width average), which have been outlined and overlaid to demonstrate that successive replicas from the same wafer possess the same geometry and features, demonstrating the functional utility of the wafer and demonstrating that the method although simple, results in the creation of reproducible, near-identical channels. While we found that the homebrew process enables the reliable reproduction of custom-manufactured channels, we note that in the initial creation of the wafers, variations in exact channel widths can occur due to the limited resolution of the inkjet printing process employed for photomask production. In addition, both the light wavelength and intensity contributed to the resulting channel geometry, with greater-intensity light producing a more complete channel, as can be seen in Figure 4.

The minimum feature resolution achievable is also significantly affected by the quality of the photomask employed, itself affected in this case by the capabilities of the printer producing the photomask transparencies, also shown in Figure 4. In this work, we utilized standard, readily available inks for both inkjet and toner-based printers for photomask transparency production and found that though similar in performance, the inkjet-printed masks tended to be more uniformly covered owing to the movement of the ink to spread out and fill any gaps, which does not occur with toner, leaving characteristic “pinholes” which adversely affects results. In this case, we found that subsequent passes through the printer could help “fill out” these patches and give more uniform shapes, such as thicker lines. Care must be taken with this, as incorrect placement can result in skewed prints that overlap. We observed that as we printed successively smaller features, there was a consequent increase in the number of “stray” ink-blots which are deposited out of place and in the intended path of the channel (as seen in Figure 4), which we wish to keep clear to allow the most light through possible so that it may selectively harden the underlying photopolymer. With our homebrew mask, the smallest reliably reproducible features were channel widths of ∼85 μm. To test the cause of this limitation, we attempted to utilize a commercially sourced chrome photomask depicting a large array of microwells of 4.4 μm diameter to investigate whether smaller features would be producible in the same photoresist if using a higher-quality photomask. We found that we were unable to develop a homebrew wafer using the dry-film photoresist, which would peel or otherwise lift off from the copper following exposure. We were not able to produce smaller features using the dry-film photoresist and that found the smallest reproducible features with this photoresist were similarly ∼85 to 100 μm.

Based on these observations, it is expected that a higher-quality printer with greater precision would enable the production of higher-quality transparency photomasks from the homebrew method allowing for smaller features to be produced. Higher-quality photomasks should also improve the uniformity of resulting channels, which would help ensure accurate flow rates and the prevention of potential pooling. To improve the resolution of resulting channels, a more “for-purpose” dry-film photoresist in place of the cheap, general-purpose photoresist could also be sought. Subsequently then, we employed the popular liquid SU8 photoresist in place of the dry film to assess whether resolution restrictions were down to the photomask and photoresist itself or due to the use of homemade equipment. Wafers were fabricated using a homemade spincoater (see the Supporting Information) to apply the resist and exposed via our improvised exposure station comprising a clamp stand holding a UV black light. While the homebrew method was capable of producing features in the sub-100 μm (width), we found that in order to produce single-digit μm features, both the chrome photomask and proper (in this instance SU8) photoresist were required. In both cases, channel heights are dependent upon the number of deposited layers. For the purposes of these rapid-prototyping experiments, the default of 50 μm (one deposited dry-film photoresist layer thickness) was used.

### 3.1 Wafer Production and Quality

The procedure parameters followed can greatly affect the resulting resolution, such as the duration of each step, where too much heat exposure can cause the photopolymer to peel/ lift from the wafer.

### 3.2 PDMS Replica Testing

**Figure 5:**
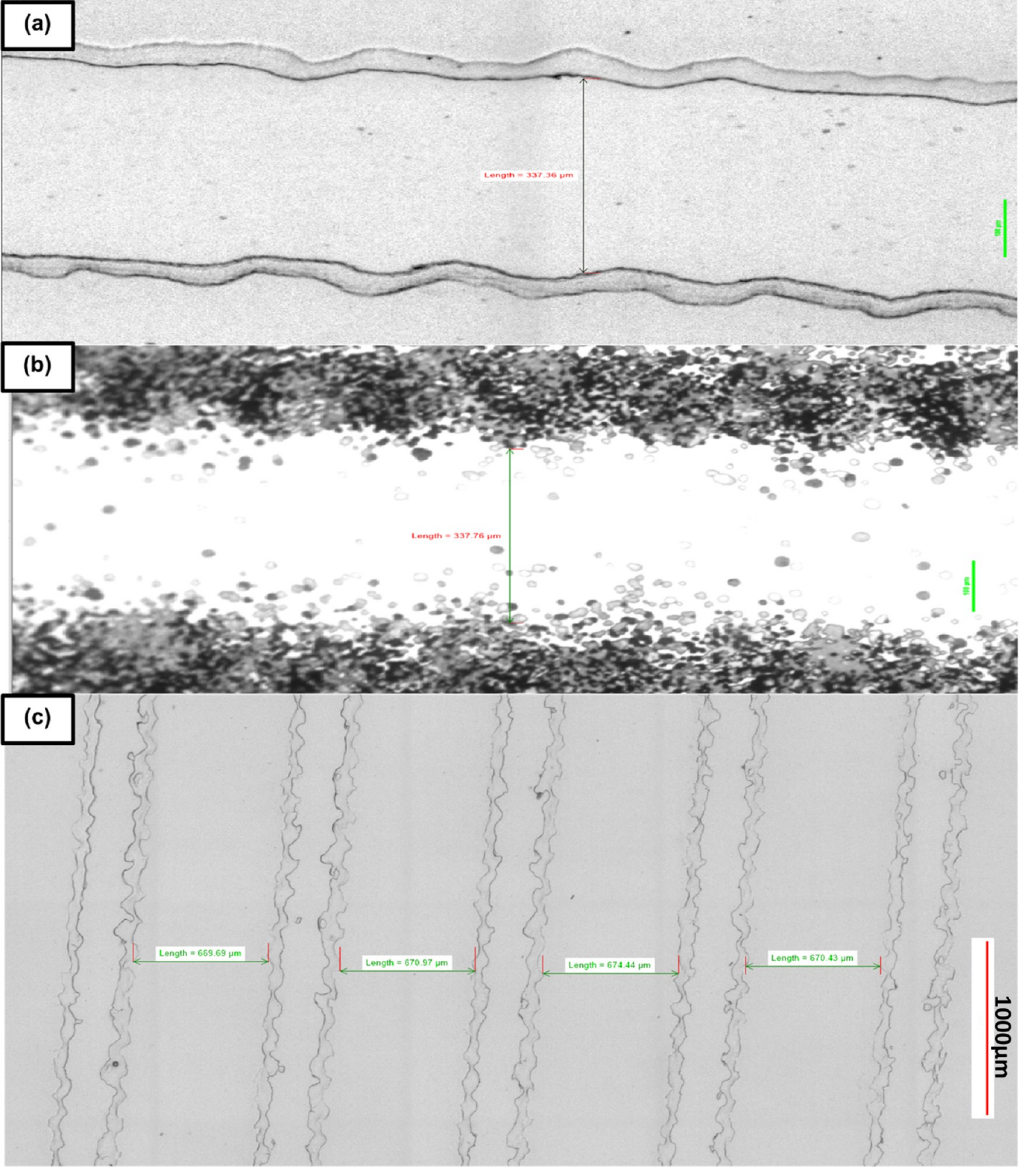
(a) Example of homebrew channel and (b) transparency photomask that was used to produce the wafer mold which the channel was molded from (channel measurement is 337.36 μm). (c) Bright-field microscope section showing the regularity and repeatability of the channels using the homebrew method (channel measurements left to right: 669.69, 670.97, 674.44, 670.43 μm.) Scale bars are 100 μm.

To test the performance of the resulting PDMS devices fabricated from our homebrew wafers, we performed simple dye tests to look for any blockages, leakages, or other kinds of obstructions. Channels were oxygen-plasma-bonded to either glass or a bottom layer of PDMS containing a microwell array. Simple suction via a connected hand-operated syringe was sufficient to induce fluid movement in all channels without any observable leakage, blockages, or obstructions, and solutions are shown moving effortlessly through the channels (see Supporting Videos in the SI). Figure 5a,b shows that the inconsistent/uneven nature of the channel walls which give the channels their “corrugated” shape is a consequence of the photomask shape and is near-perfectly reproduced in separate resulting replicas, which can be overlapped to demonstrate faithfulness and repeatability of replication, as depicted with Figure 4. Additionally, these so-called “corrugated walls” have no bearing on the channels resulting functionality, and channels are repeatedly flat and uniform, as is evident in Figure 5c, with no visible particle or otherwise potentially obstruction-like structures contained within.

**Figure 6:**
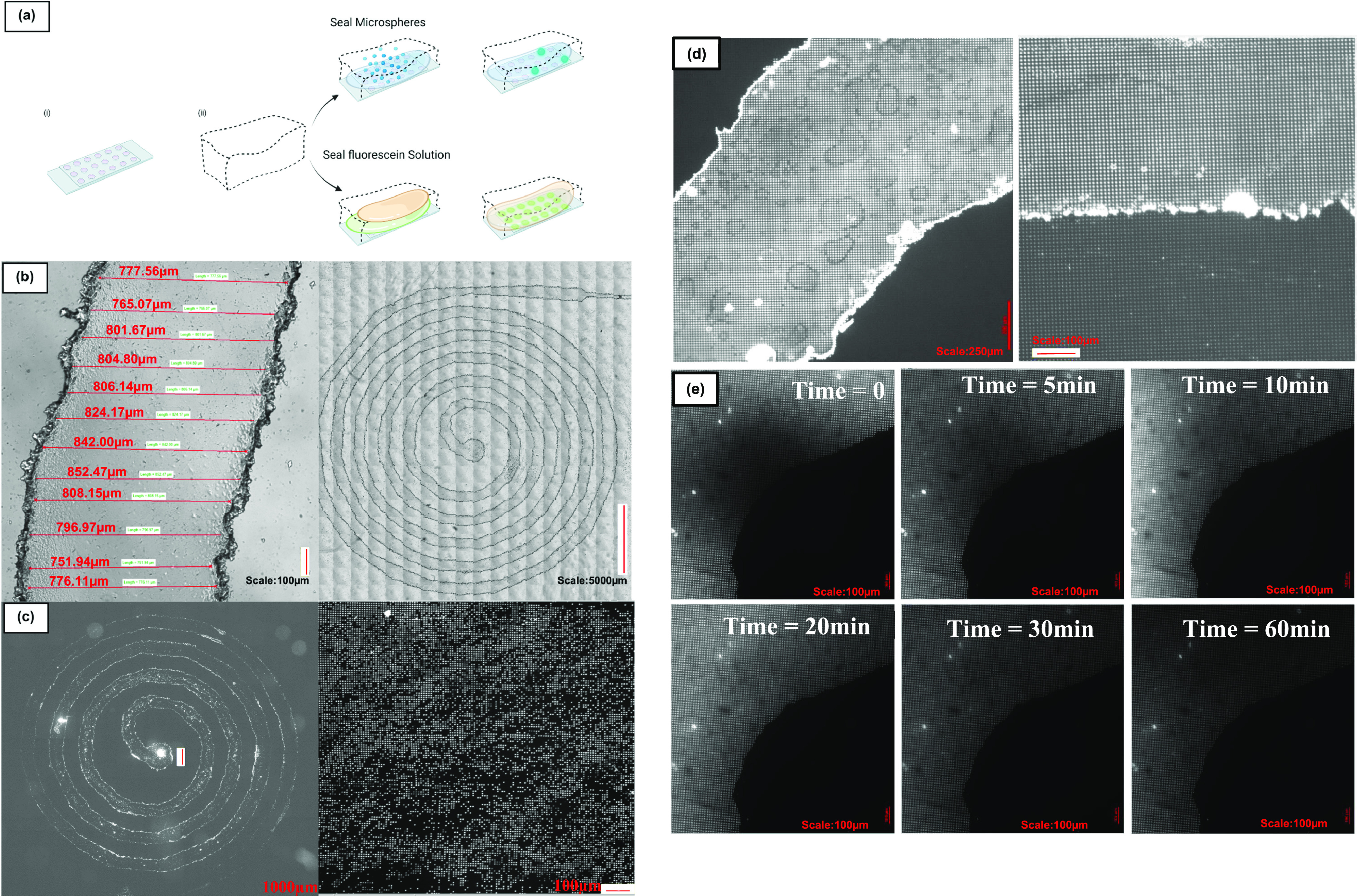
(A) Schematic of the first approach to functionally testing homebrew channel integrity; microwells produced using chrome photomask (i) served as planar array of microchambers to enclose with a homebrew channel (ii) and subsequently introduce fluorescent microspheres or a fluorescein and mineral oil solution to. (B) Close-up and zoomed-out examples of spiral channels made using the homebrew method, showing reasonably even widths and formation of the intended, regular patterned spiral geometry. (C) Zoomed-out homebrew channel used to seed fluorescent microspheres underway and close-up showing high final seeding efficiency within the enclosed channel. (D) Sectional images of the homebrew channel enclosing fluorescein into individual microwells via mineral oil introduction into the channel via simple manual pipetting. (C, D) Channel is functional when bonded on top of an array of microwells; it can be used to achieve high seeding via delivery of a dispersed solution of fluorescent microspheres into the microwells or enclose individual microwells with aqueous solutions, which could have applications in high-throughput single-cell studies. All wells are nonpreferentially filled, which would not be the case if the interior of the channel was debilitatingly uneven, demonstrating that the channels can be used functionally in experimentation, and furthermore, no pooling is observed. (E) Time lapse of homebrew spiral channel bonded on top of microwells and used to fill and seal individual microwells with fluorescein and mineral oil—here, it is observable that the wells are all filled and sealed inside an intact channel—as evidenced by no apparent leakage of fluorescent solution. Scale bars are 100 μm.

**Figure 7:**
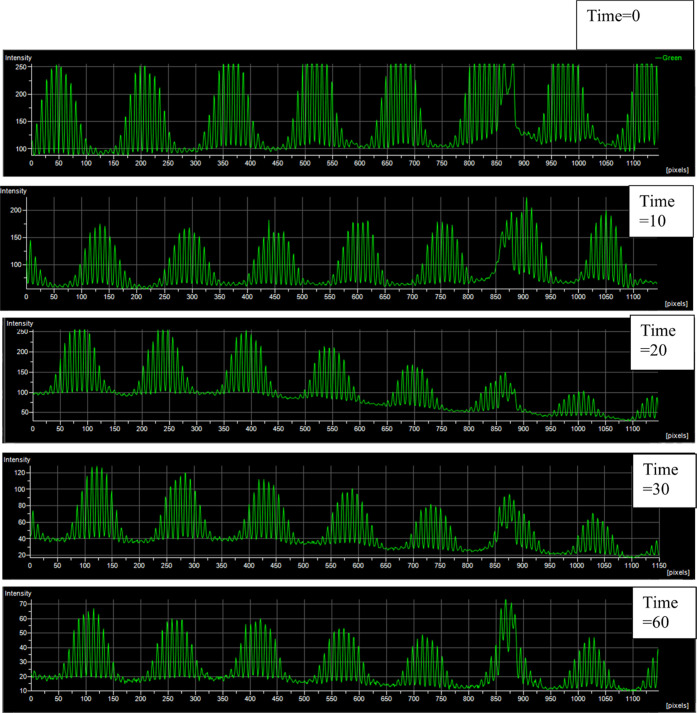
Intensity profile across time lapse of fluorescein-sealed homebrew spiral channel. During the time-lapse imaging of the channels containing fluorescein and mineral oil, intensity profile measurements were made at time intervals; time = 0, 10, 20, 30, and 60 min in order to evaluate the change in intensity over time and highlight whether the channels allowed the solution to fill and seal the wells, without leakage or failure to homogeneously seal due to rough, uneven surface topology. Discretization of the peaks shows a regular surface profile with a regular pattern throughout the time lapse, indicative of a discretized surface comprised of regular arrays of wells containing trapped fluorescein. A characteristic drop is reflected equally across all peaks as the regular excitation of the fluorescein causes the fluorescence emission to decline over time as photobleaching occurs and the fluorophores are no longer promoted to an excited state. Such a regular, contained pattern of intensity changing in unison indicates that the channels serve their functional purpose, facilitating movement of fluorescein and then mineral oil across the surface of the microwell arrays and then containing sealed solution within. Time is presented in minutes.

We next utilized the produced microwell arrays that were produced via SU8/chrome photomask to evaluate the homebrew channel integrity through bonding of homebrew channels on top of the well arrays to provide an enclosed array of microwells, which could then be seeded with microspheres or sealed with fluorescein and mineral oil in order to evaluate the channel’s functionality. As shown in Figure 6, the channels can be utilized to carry a dispersed solution of microspheres (Figure 6C), which consequently seed the wells only in enclosed regions, or effectively seal an array of microwells with fluorescein solution (Figure 6D) with no observed movement between wells or leakage out of the channel enclosure, as demonstrated through time-lapse imaging (Figure 6E) and light intensity measurements (Figure 7). Both seeding and sealing were achieved robustly across the cross section of the channels, indicating that the channels were not debilitatingly uneven on the inside or full of foreign structures, which would perturb entry into sections of microwells. Finally, corrugated or uneven channel walls which are functional are not unusual when considering the prevalence in nature for similar structures within biological vessels such as intestines.

**Figure 8:**
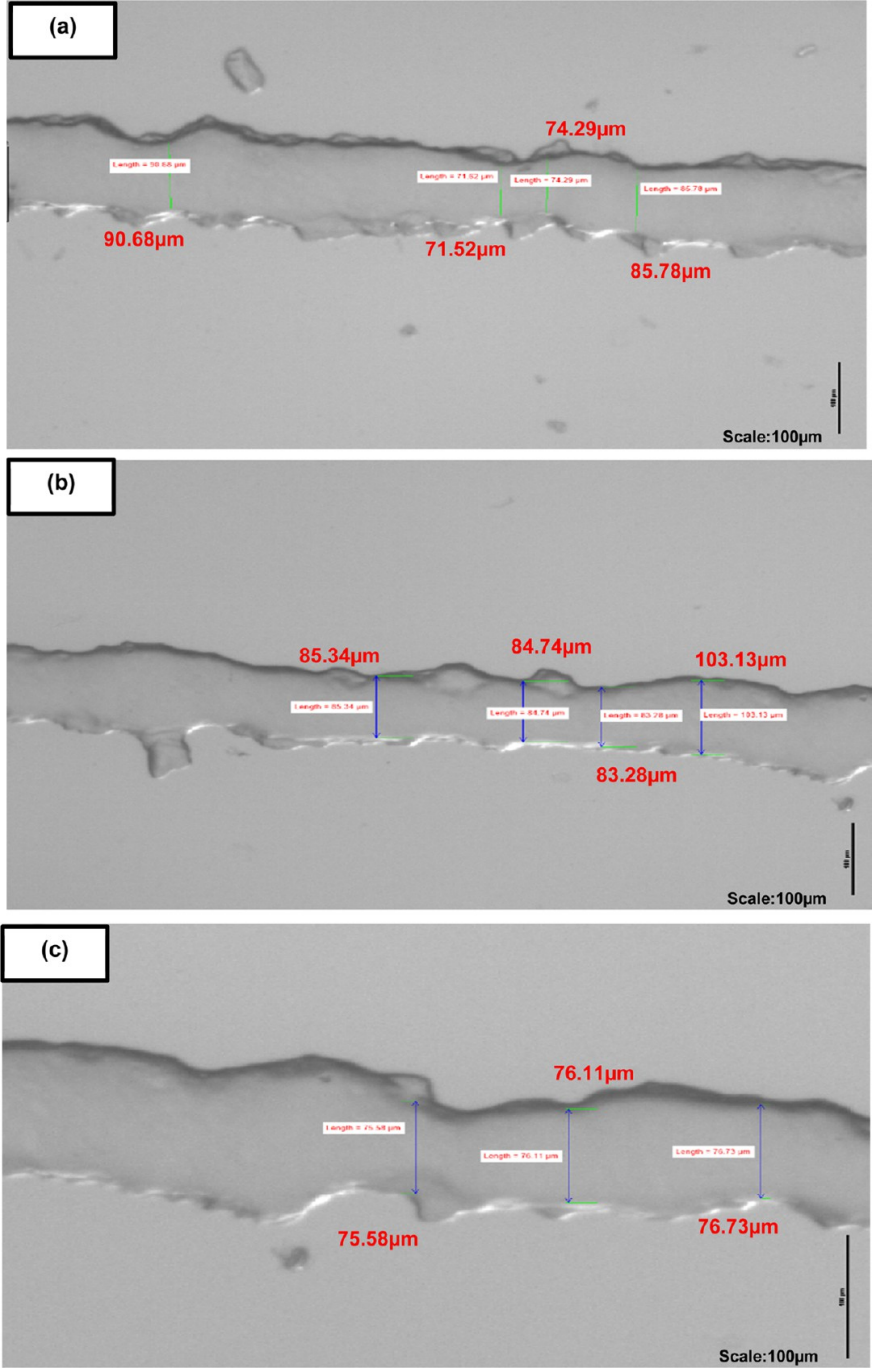
Smallest reproducible channels via the homebrew method. Repeated production of wafers and subsequent replica molding in PDMS confirmed that the smallest readily reproducible features using the homebrew method were in the range of 75–100 μm, with a 50 μm depth representing one deposited layer of the dry-film photoresist. Scale bars are 100 μm. Measurements are (a) 90.68, 71.58, 74.29, 85.78 μm, (b) 85.34, 84.74, 83.28, 103.13 μm, (c) 75.58, 76.11, 76.73 μm.

Production of homebrew wafers and subsequent replica molding from the wafers confirmed that larger feature sizes on the order of 200–1000 μm were most easily and repeatedly reproduced (Figures 5c and 8) with the smallest repeatedly reproducible channel dimensions (Figure 8) being 50 μm height (representing one single deposited dry-film photoresist layer) and channel widths of 75–100 μm. Comparisons between Figures 5c and 8 highlight that the repeatable uniformity of channels over several replicas is compromised when dropping below <100 μm in feature widths, at which point more variation between replicates of the same patterns is observed.

We next compared our homebrew approach to the most popular current rapid-prototyping DIY microfluidic approach, which is stereolithography-based 3D printing (Table 3). Photolithography and soft lithography, along with SLA 3D printing, are popular techniques used for microfluidics mold and chip production. Briefly, SLA 3D printing utilizes photopolymerizable resins in tandem with a projection of a changing series of pixels in particular patterns in order to cure sequential layers of resin into desired three-dimensional shapes. Each method has its advantages. The advent of 3D printing has opened up new possibilities for the field of microfluidics. The flexibility in design from a simple 3D CAD file empowers researchers to explore innovative and tailored solutions for specific applications. 3D printing enables fast and cost-effective prototyping of microfluidic devices, researchers can design and print multiple iterations of microfluidic devices in a relatively short timeframe, facilitating the optimization of device parameters and performance.^22^ This accelerated prototyping process saves time, reduces costs, and enables quick iterations in the development cycle. 3D printing allows for the integration of complex features and functionalities within a single microfluidic device. It enables the fabrication of intricate channels, chambers, valves, mixers, and other microstructures in a single print, simplifying the overall device assembly.

**Table 3:** Advantages and Disadvantages of Stereolithography-Based 3D Printing for Production of Master Molds

advantages	limitations
**design freedom**: SLA 3D printing allows for the creation of complex, customized mold geometries that are difficult to achieve with other techniques.	**material limitations:** the choice of materials for SLA is limited compared to other techniques, which can restrict the range of applications.
**high resolution**: SLA can achieve high resolution, typically in the range of tens to a few hundred microns, depending on the printer and resin used.	**limited scalability**: although SLA offers faster prototyping, scaling up for mass production can be time-consuming and expensive due to the layer-by-layer printing process.
**rapid prototyping**: SLA enables quick prototyping with shorter lead times compared to traditional mold fabrication techniques.	postprocessing requirements: SLA-printed molds may require additional postprocessing steps, such as cleaning and curing, which can add complexity to the overall workflow.

This integration of multiple functionalities within a single device enhances the complexity and capabilities of microfluidic systems. 3D printing offers customization options for microfluidic devices, allowing researchers to tailor the device specifications to their specific needs. Customization includes the adjustment of channel dimensions, geometries, and layouts, as well as the incorporation of specific sensor integration or sample handling features. Additionally, 3D printing enables easy scalability, as designs can be easily modified and printed in different sizes or quantities to accommodate various experimental requirements. 3D printing provides a wide range of material options for microfluidic device fabrication. Depending on the desired properties, researchers can choose from various materials such as thermoplastics, elastomers, hydrogels, or biocompatible polymers. This material versatility enables the fabrication of microfluidic devices with tailored characteristics, such as optical transparency, mechanical flexibility, or chemical resistance, to suit specific applications. 3D printing allows for the fabrication of monolithic microfluidic devices without the need for complex assembly or bonding processes. Traditional fabrication techniques often require multiple steps for device assembly, including bonding of different layers or components. With 3D printing, complex microfluidic devices can be printed as a single monolithic structure, eliminating the need for additional assembly steps and improving device robustness and reliability. 3D printing has made microfluidics more accessible and affordable, especially for research laboratories and academic institutions.^23^

The reduced cost of 3D printers and readily available open-source designs have democratized microfluidic device fabrication. Researchers can now create their microfluidic devices in-house, reducing the reliance on specialized fabrication facilities or expensive commercial products. 3D printing enables on-demand production of microfluidic devices. Researchers can quickly design and print microfluidic devices as needed, eliminating the need for large-scale manufacturing and storage. This flexibility in production allows for iterative design improvements and easy adaptation to changing experimental requirements facilitating integration with other technologies, for example, sensors, electrodes, optics, or electronic components. These can be integrated directly into the 3D-printed microfluidic device during fabrication, and this seamless integration enhances the functionality and versatility of microfluidic systems for various applications, such as biosensing, cell culture, or point-of-care diagnostics.

**Figure 9:**
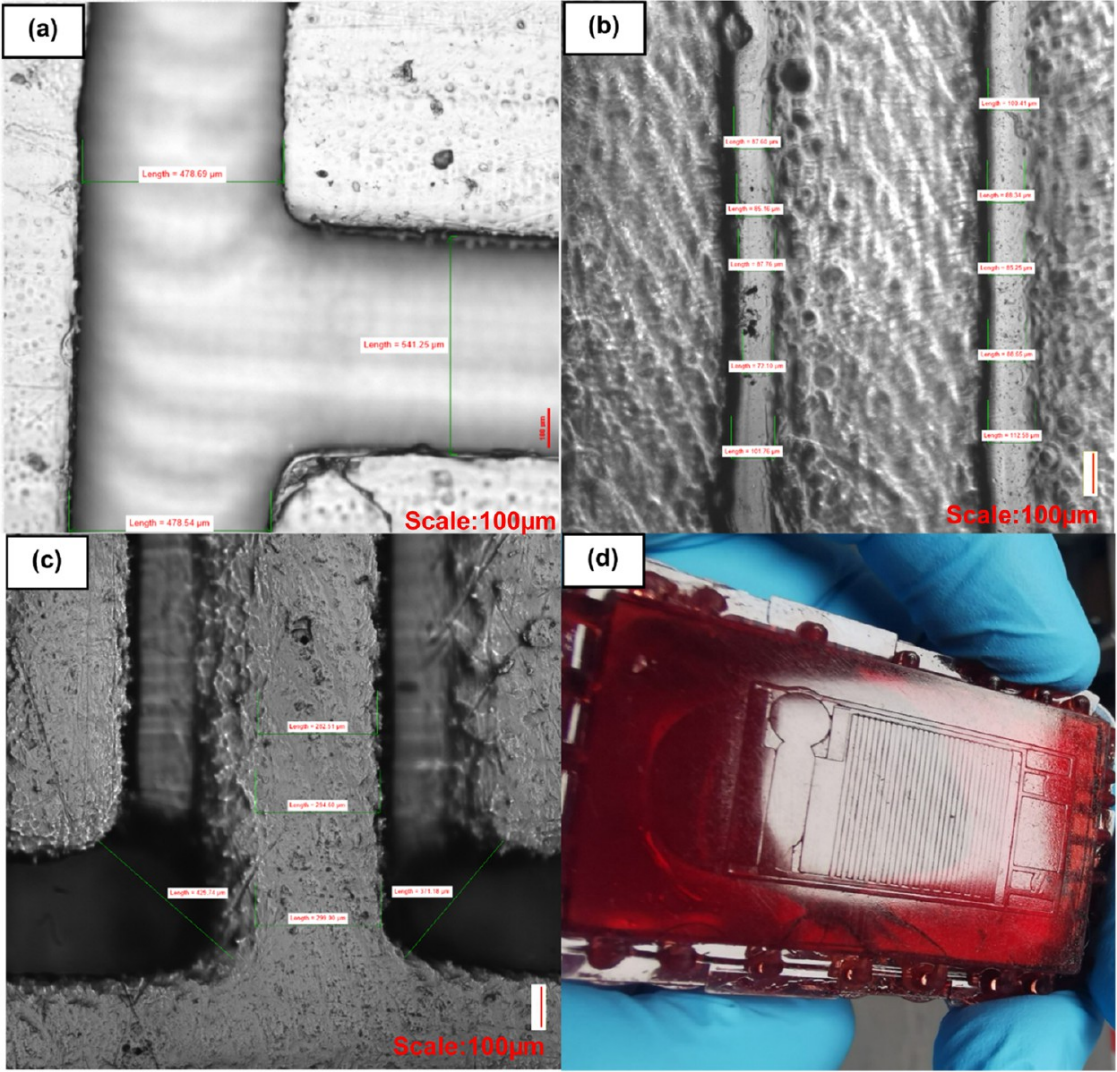
(a, b) Example of 3D-printed channel replicated in PDMS. (c) Surface of SLA mold. (d) Zoomed-in SLA 3D-printed master mold that the PDMS was molded from. Irregular debris or artifacts are visible inside the channels in (b). Measurements are (a) 478.69, 541.25, 478.54 μm, (b) 87.60, 85.16, 87.76, 72.10, 101.76, 100.41, 88.34, 85.25, 88.55, 112.58 μm, and (c) 429.74, 282.51, 294.60, 299, 371.18 μm.

On balance, traditional photolithography provides high resolution and scalability but is expensive and limited to planar molds. SLA 3D printing provides design freedom, high resolution, and rapid prototyping, but material limitations and scalability challenges exist. The choice of technique depends on the specific requirements of the microfluidics application, considering factors such as resolution, cost, mold complexity, and scalability needs. We found that although in principle SLA 3D printing could be employed to produce microfluidic master molds (Figure 9), their resolution was limited to ∼450 μm for smooth finished/properly formed channels (Figure 9a). In addition, their downstream utility was limited owing to the surface roughness of the mold, which was transferred to the replica in the soft lithography molding process, and this appeared to worsen as feature resolution became smaller. This surface roughness (evident in Figure 9b,c) compromised the ability of the channel to form a durable seal to either glass or PDMS, preventing the integration of this component with other materials toward multimaterial composite devices. This is problematic when making devices which involve channel enclosure to facilitate entry across arrays of microwells as presented previously with the homebrew method—the SLA 3D-printed mold was unable to achieve this. Delamination was observed at room temperature by simply handling the chips, confirming their lack of suitability as a functional channel to enclose microchambers.

**Figure 10:**
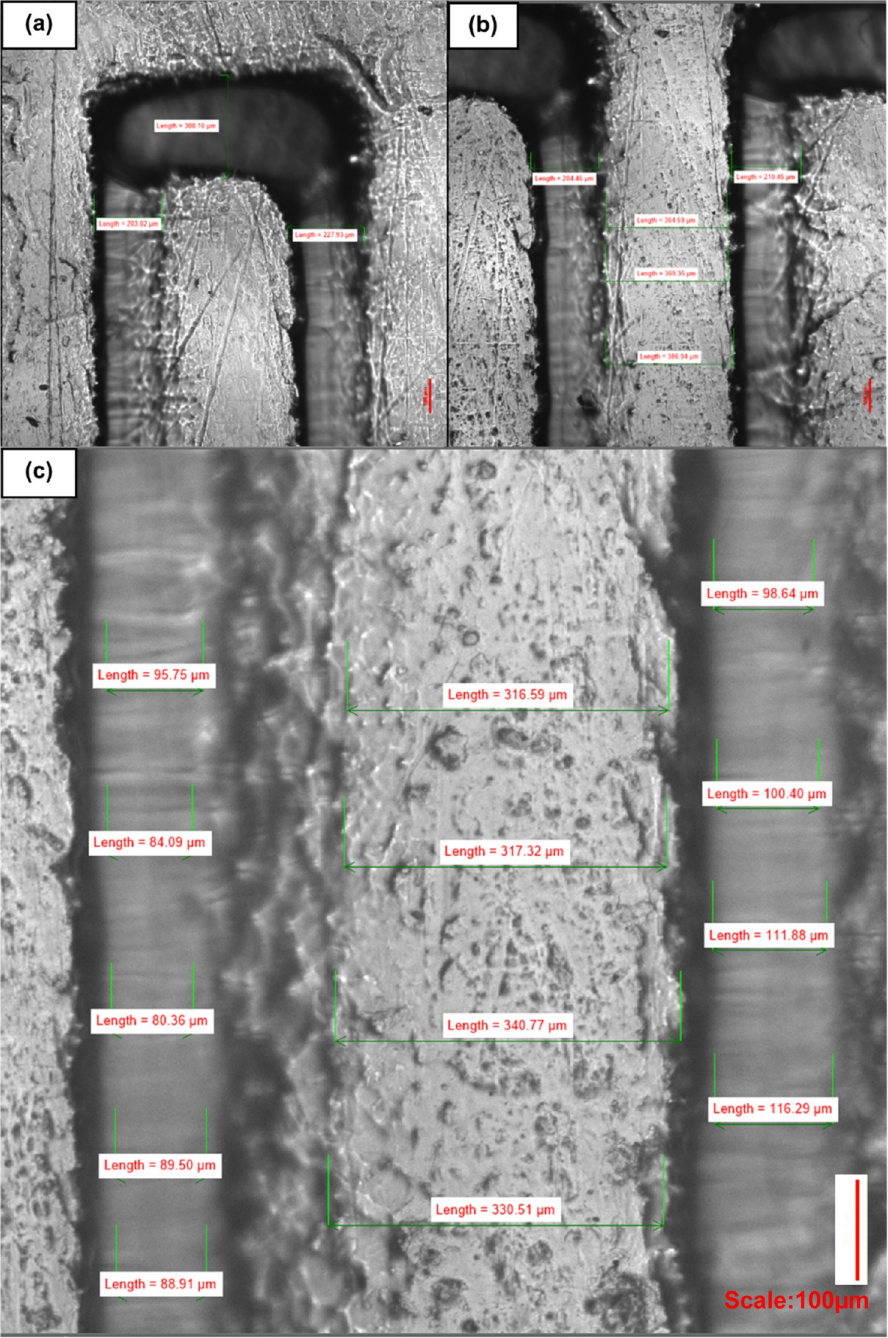
Closer look at the SLA 3D mold showing an uneven and rough surface, which is imprinted upon the PDMS and results in an uneven PDMS replica surface that impedes downstream applications due to the inability to securely bond the channels to glass or other PDMS. Measurements are (a) 203.02, 300.10, 227.93 μm, (b) 204.46, 364.59, 369.35, 386.94, 210.45 μm, and (c) 95.75, 84.09, 80.36, 89.50, 88.91, 316.59, 317.32, 340.77, 330.51 μm.

**Figure 11:**
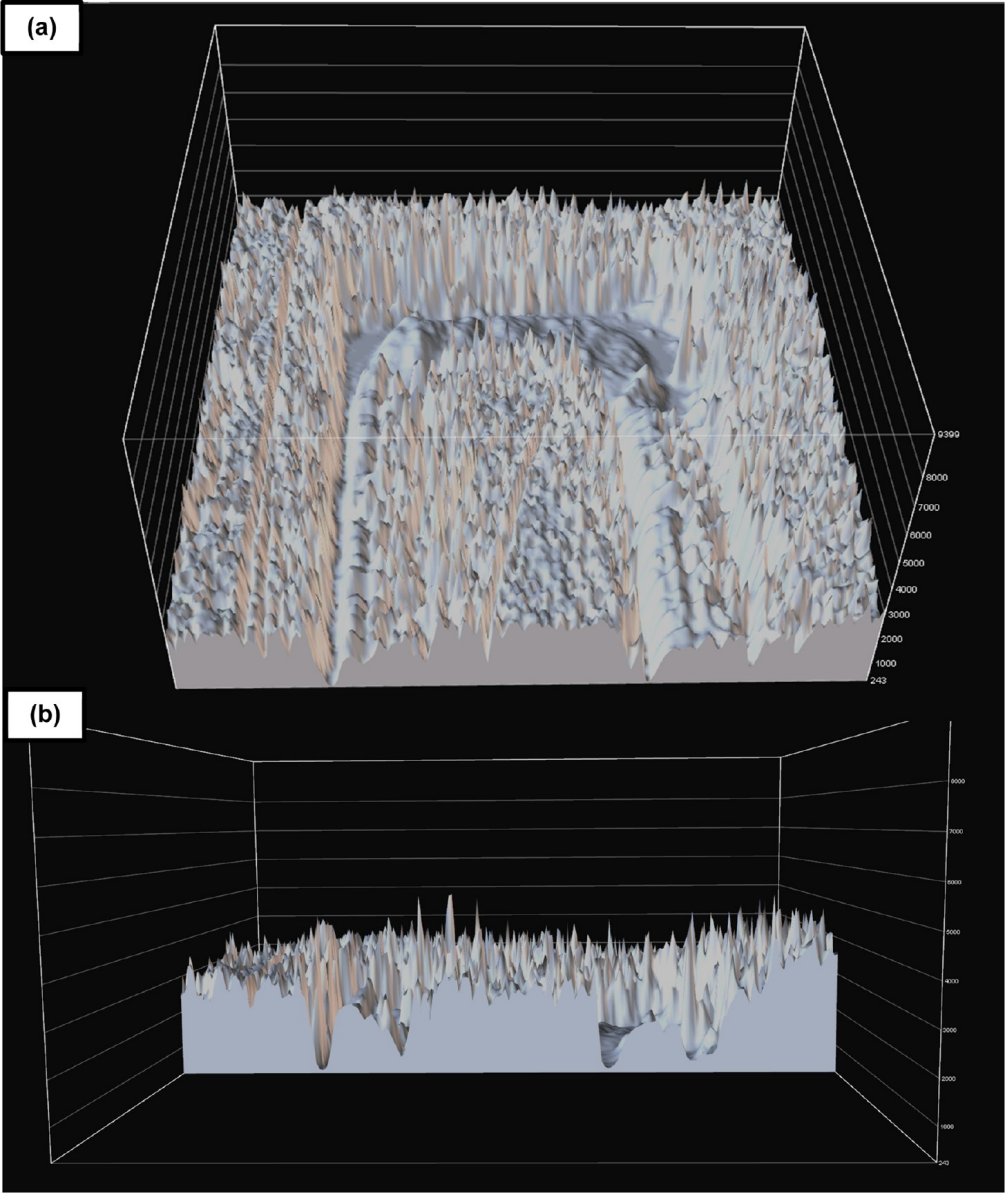
(a) Top-view surface intensity profile of SLA 3D-printed straight channels seen in Figure 9 based on scattered light from optical micrographs and (b) cross section. The SLA 3D-printed master mold possessed a total thickness of 3000 μm. Positive features were 300 μm in height, which represented the lowest layer thickness that resulted in reproducible features.

**Figure 12:**
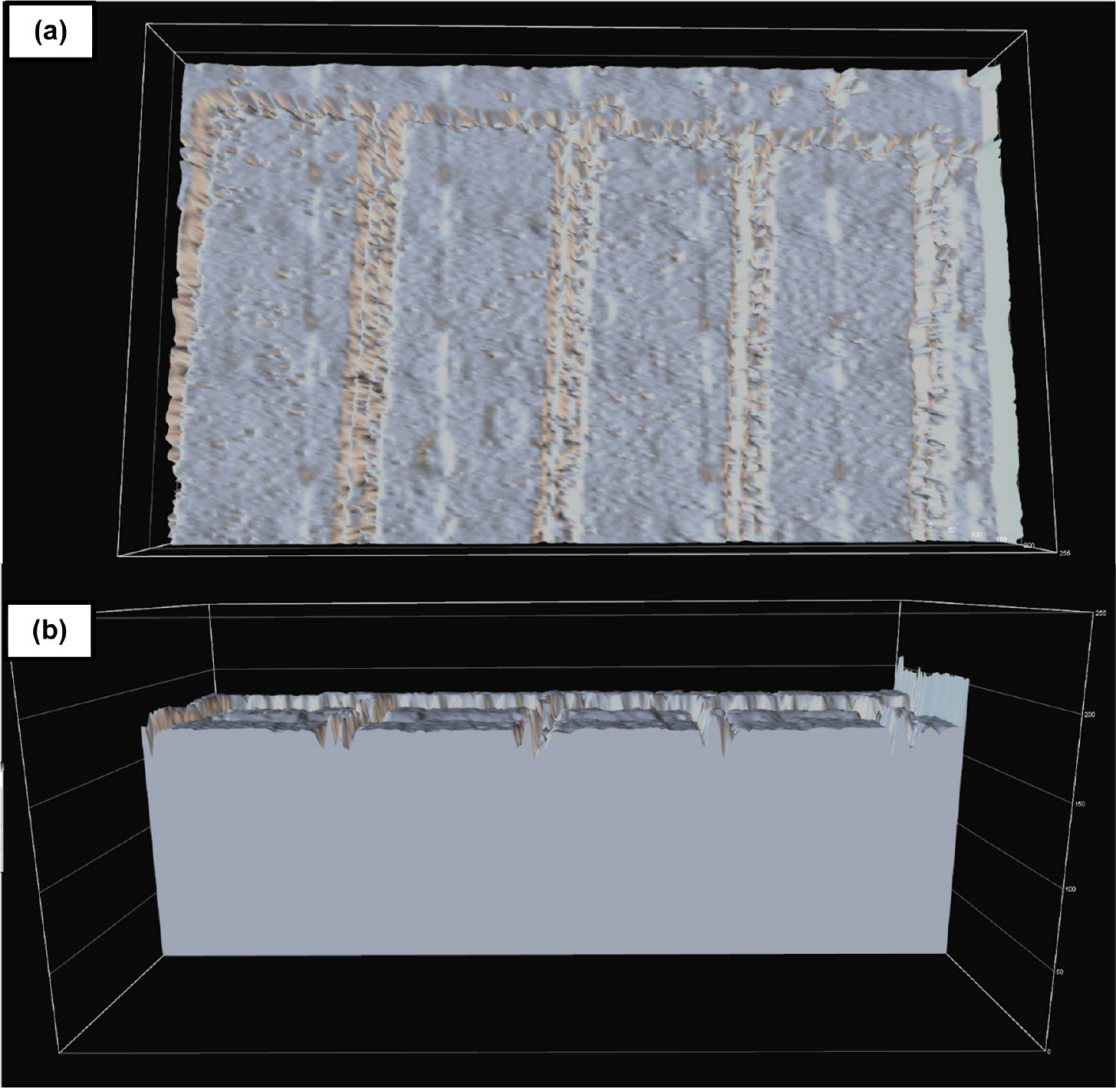
(a) Top-view surface intensity profile of homebrew straight channels seen in Figure 5C based on scattered light from optical micrographs and (b) cross section. The Homebrew master mold possessed a total thickness of 50 μm, which was the height afforded by the lamination of one single sheet of the dry-film photoresist.

Although overall channel geometry can be observed (Figure 10), it is known that PDMS chips made from SLA 3D prints can contain rough surfaces, requiring some form of surface characterization.^23^ On closer inspection (Figure 11) via a surface intensity plot conducted using NIS-Elements software from collected micrographs, variability is high for SLA 3D-printed channel geometries and surface roughness is similarly evidently high. By comparison, a surface intensity plot conducted on the same channel patterns produced via the homebrew method (Figure 12) showed significantly flatter, more uniform topographical space. With regard to the SLA 3D-print-produced channels, such surface roughness not only contributes to a weaker potential bonding in multilayer structures when used as a mold but can also make cleaning the molds tricky, and residual leaching from the photopolymer resin can result in curing inhibition, limiting the usefulness of such a mold. In addition, difficulty in cleaning such a mold owing to the uneven surface topology can limit the potential uses of SLA 3D-printed components in life sciences experimentation that requires usage of sensitive material such as cells—whereby residual resin leakage could prove harmful to the cells and adversely affect the sample and/or the conducted experiment. Furthermore, such reporting of curing inhibition due to photopolymer resins, limiting their use in microfluidics has been well reported in the literature.^24^

We found that compared with SLA 3D printing for mold production, our homebrew method was greatly simpler, quicker, and lower cost to implement, with the added benefit of no additional complexities regarding downstream curing inhibition, unlike with 3D-printed molds from cured photopolymer resin.

**Figure 13:**
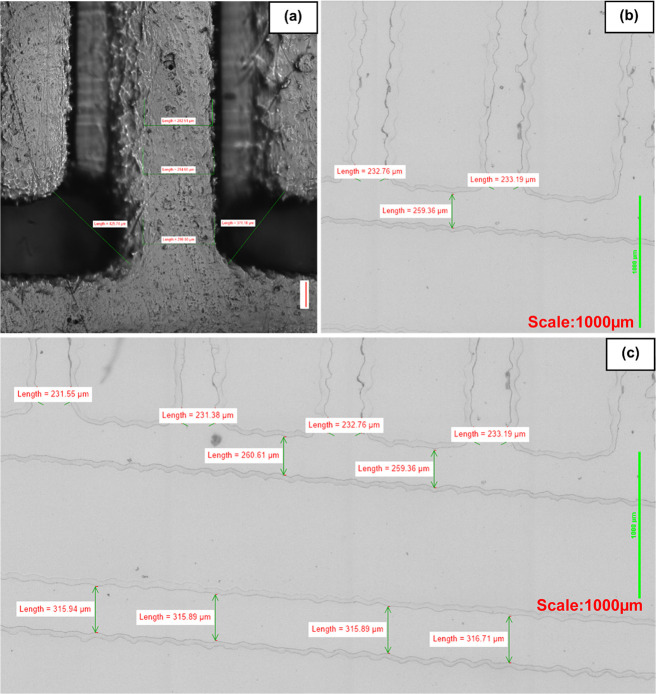
Comparison between 3D-printed (a) and homebrew-produced channels (b, c). Homebrew channels produced a flatter, more uniform surface and lower variation in dimensions than the 3D-printed counterpart, whose rough surface resulted in greater variability in channel walls. The consequent surface roughness of the 3D print was replicated by the PDMS chip which consequently failed to plasma bond to glass due to the uneven surface, (a) 282.51, 294.60, 429.74, 299, 371.18 μm, and (b) 232.76, 259.36, 233.19 μm, and (c) 231.55, 231.38, 260.61, 232.76, 259.36, 233.19, 315.94, 315.89, 315.89, 316.71 μm.

**Figure 14:**
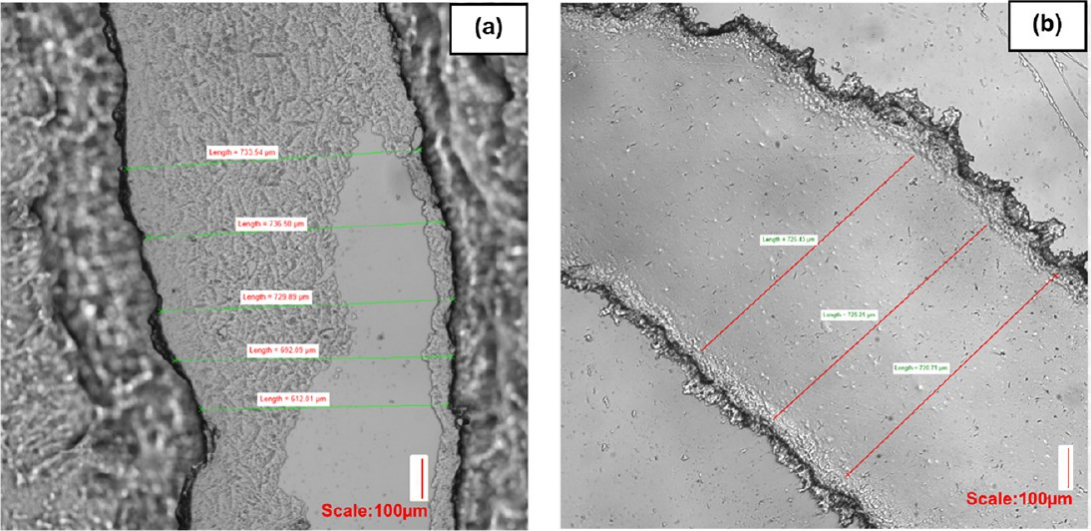
Comparison between spiral channel from SLA 3D print (a) and the same channel produced using the homebrew method (b). While (b) was imaged from above to show more clearly the dimensionality of the channel, (a) could only be imaged from underneath due to a low opacity of the mold from above as a result of the rough surface replicated by the chip causing light scattering and making a clean focus difficult. By comparison, the SLA mold results in a rougher and more uneven surface with respect to the homebrew approach both inside and on the outside surrounding the channel. Small artifacts present in (b) were merely lingering free particulates present on the outside of the channel prior to rinsing, which are readily removed with a simple IPA rinse, leaving cleaner-looking channels, such as those shown in Figure 11c. Measurements are (a) 733.54, 736.50, 729.89, 692.09, 612.01 μm and (b) 725.43, 725.25, 720.71 μm.

We found that channels made using the SLA 3D-printed molds were unable to form a strong seal to glass or PDMS when oxygen-plasma-bonded and would often delaminate from mere handling of the device, thus rendering the molds unsuitable for use in producing multidimensional chips. The best SLA 3D-produced channels were still weakly bonded, and we found that upon introduction of test microsphere solution through channels bonded on-top of microwells that solution leaked out of the defined channels immediately, confirming that they were not sealed (Figure S4). Figure 13b,c shows that the homebrew method was capable of producing the same regular rectangular microfluidic channels from the same pattern with clear, clean finishes and no readily observable, obvious surface roughness, which was the case for the same pattern produced via 3D printing. We found that channel dimensions were consistently reproducible and well-defined considering the simplicity and “low-tech” nature of the method. We note that, in addition to the rectangular channels, the homebrew method also produced flatter, more uniform spiral channels (Figure 14b) with no obvious surrounding surface roughness, which was not the case for the same spiral design produced from an SLA 3D print (Figure 14a).

We note in general, however, that as feature sizes become smaller, occurrences of small artifacts in or around the channel become more readily apparent, which we believe to be consistent with our earlier observations that this is a consequence of the limited resolution of the low-cost transparency masks, whereby the artifacts are likely a product of stray ink blots affecting the uniformity of the photoresist selective hardening and dissolution by development. We have found that with channels in the range of 100 μm, these occasional small artifacts had no bearing on resulting functionality, but for more uniform results, higher-quality photoresist or higher-resolution printed photomasks could be employed to circumvent this issue.

## 4 Conclusions

In this paper, we provide a simple, quick, low-cost means of rapidly prototyping microfluidic devices from scratch utilizing only readily available, easily accessible materials. With this method, we show the repeatable, reliable fabrication of both rectangular and spiral channels, as well as the production of sub-100 μm width features. We show that our method is more readily useful and able to produce the desired features with greater ease than can be achieved using SLA 3D printing.

While the overall fabrication quality and resolution cannot be compared with high-end photolithography, we feel that the homebrew approach to photolithography provides a very applicable, low barrier to entry for researchers looking to experiment and rapidly prototype ideas to validate proofs of concept before devoting expense to higher-quality, higher-cost commercial production. The homebrew approach can improve the rapid-prototyping process, reduce costs, improve overall efficiency, and provide a simple solution to those researchers requiring low-resolution microfluidic devices.

## Supporting Information Available

Video showing process of Homebrew Photolithography (Video 1) (MP4)
Simple suction via a connected hand-operated syringe was sufficient to induce fluid movement in all channels without any observable leakage, blockages, or obstructions, and solutions are shown moving effortlessly through the channels (Video 2) (MP4)
Building a homemade spincoater for application of liquid photoresist to silicon wafers (Figure S1); CAD design and resulting channel molded in PDMS from homebrew wafer of serpentine design (Figure S2); SLA 3D-printed rectangular channel pattern mold and PDMS replica (Figure S3); and composite fluorescence micrographs showing failure to contain microsphere solution of channels produced from 3D-printed mold (Figure S4) (PDF)

## Notes

All photo and micrograph images and data contained in this manuscript were generated by the authors.
