# Supplementary material for: Homebrew Photolithography for the Rapid and Low-Cost, “Do It Yourself” Prototyping of Microfluidic Devices
Source: ACS Omega. 2023 Sep 15;8(38):35393–409. doi: 10.1021/acsomega.3c05544 (PMC10535262; doi:10.1021/acsomega.3c05544)
Supplement: Supplementary file 3 — ao3c05544_si_003.pdf [file ao3c05544_si_003.pdf]

SUPPLEMENTARY INFORMATION

Homebrew Photolithography for the rapid and low-cost, 'Do it Yourself' prototyping of microfluidic devices

Daniel Todd, Natalio Krasnogor*

Interdisciplinary Computing and Complex BioSystems (ICOS, Newcastle University, United Kingdom,
Email: Natalio.krasnogor@newcastle.ac.uk

Supplementary

Spincoater assembly

The home-made spincoater was built as follows: To make the chuck which the wafer will sit upon, a hook and loop rotary polisher backing pad for a rotary sander was drilled in the centre of the disc and widened with a penknife to roughly the size of a 20 pence piece to accommodate a metal self-locking nut, which was then super-glued into place. Additionally, a glass petri-dish can then be fixed in place on-top with double-sided tape. A mini permanent magnet DC motor (12V, 3500RPM) was wired to a DC, 12V, PWM DC motor speed controller, adjustable stepless governor regulator by feeding the wires into the designated terminals and securing in place with a Phillips screwdriver. For the motor, the red(live wire) and the black wire from the motor were attached using a Phillips screwdriver to the terminals marked Motor + and Motor - respectively.
For the power-supply, two SATA cables (red & black) were stripped and secured into the terminals of the 100-240V AC to 12V DC 1Amp(1000mA) switching power supply adapter. Once the adaptor ends were secured into the terminals, the other ends were similarly secured to the DC motor speed-controller using a Phillips head screwdriver, in the ports designated power +(red wire) and power - (black-wire) respectively.
For a suitable housing container, a suitably large hole to accommodate the shaft of the DC motor was drilled and cut into the lid of a polypropylene storage box. A rectangular window was cut out to the size of the motor speed controller which was fed through and fixed into place with adhesive tape, holes to feed through and fix in place the power and speed dial were also cut into the front. The motor itself was secured inside the box in the centre with thick, double-sided adhesive.

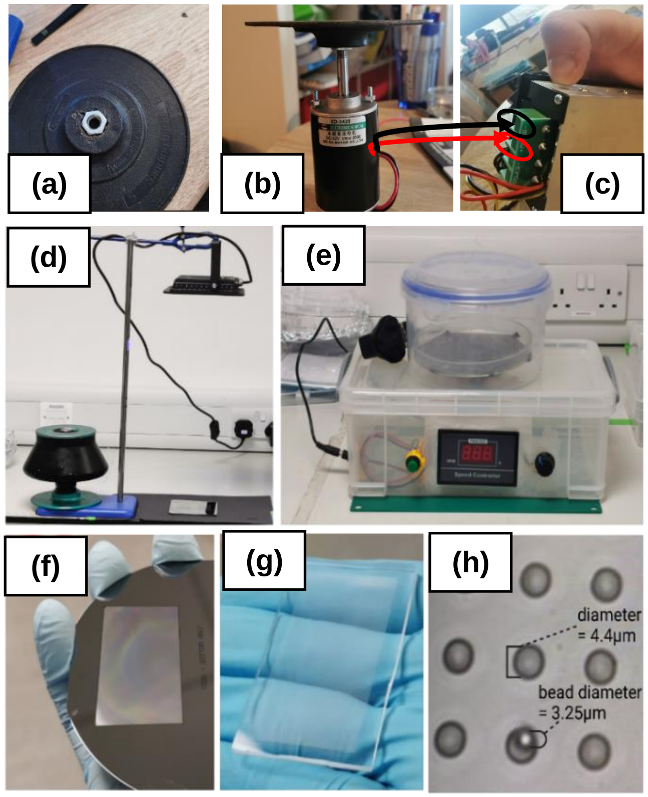

Figure S1. Building a home-made spincoater for application of liquid photoresist to silicon wafers. (***a***) hook and loop rotary polisher backing pad for a rotary sander with a locking nut fixed into it to serve as the chuck for wafer placement, which is fixed into place on a DC motor(***b***), which can be controlled through wiring to a DC motor speed controller (***c***), exposure station (***d***) & (***e***) home-made spincoater, (***f***) wafer produced from SU8 and chrome photomask which can be used to produce mold PDMS(***g***) containing microwells of 4.4μm(***h***). All Images were taken by the Authors.

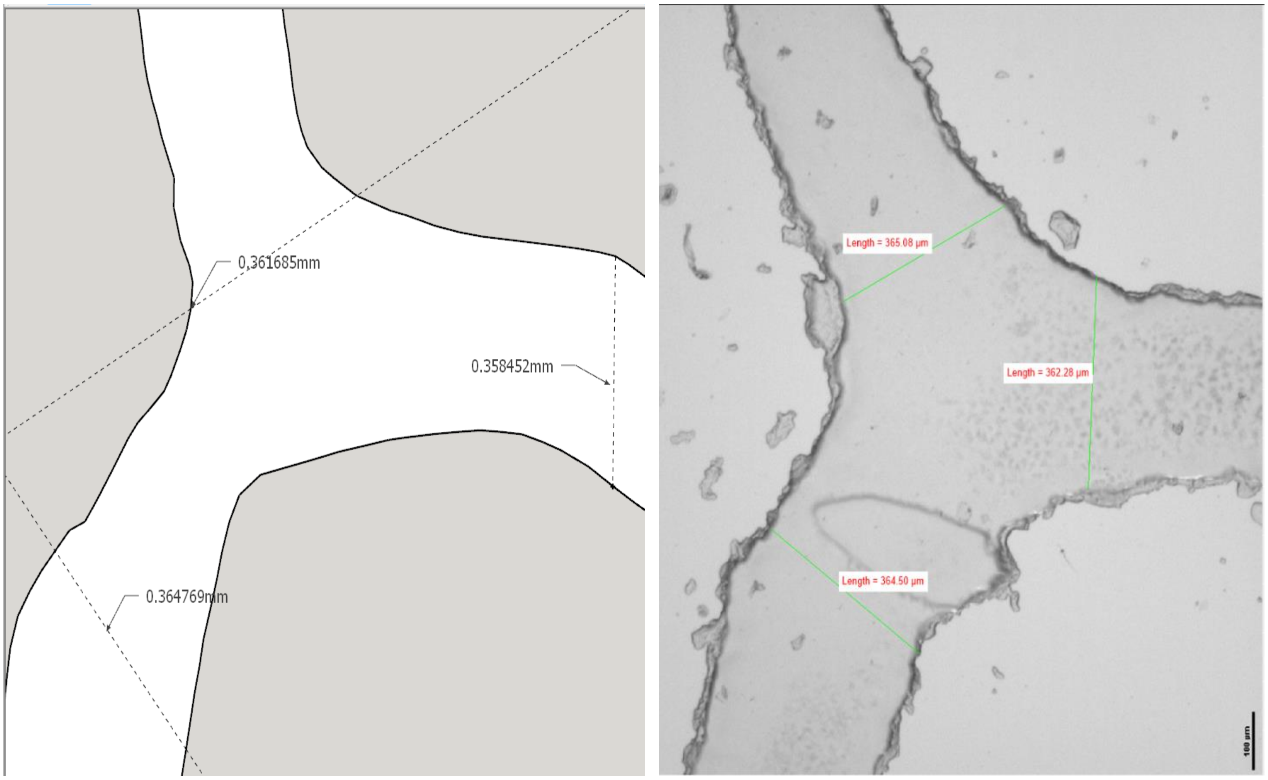

Figure S2. CAD design and resulting channel moulded in PDMS from Homebrew Wafer of Serpentine design

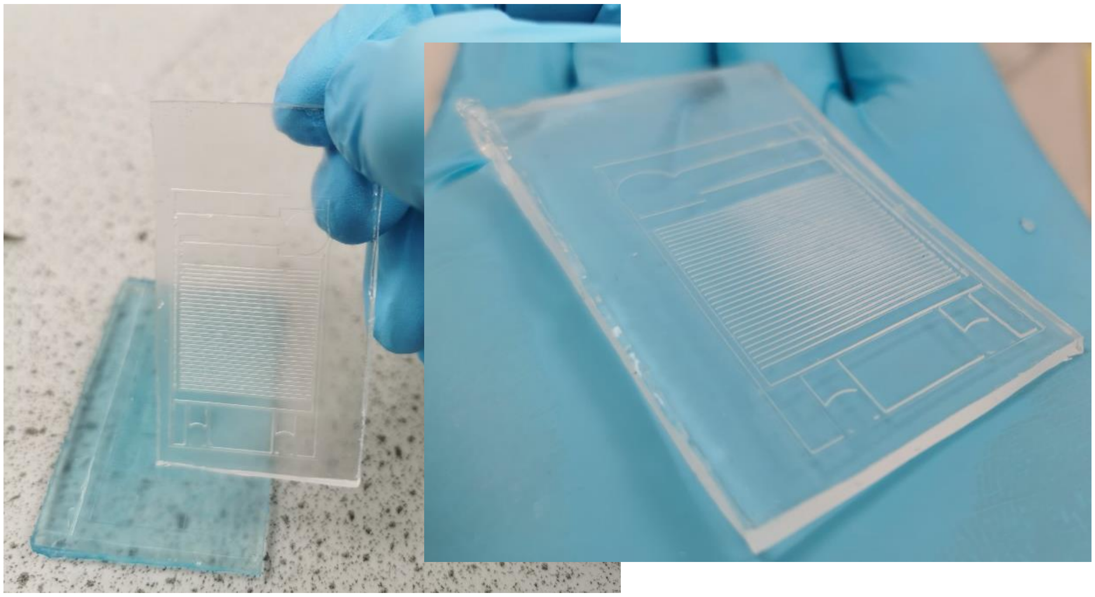

Figure S3. SLA 3D printed rectangular channel pattern mould & PDMS replica.

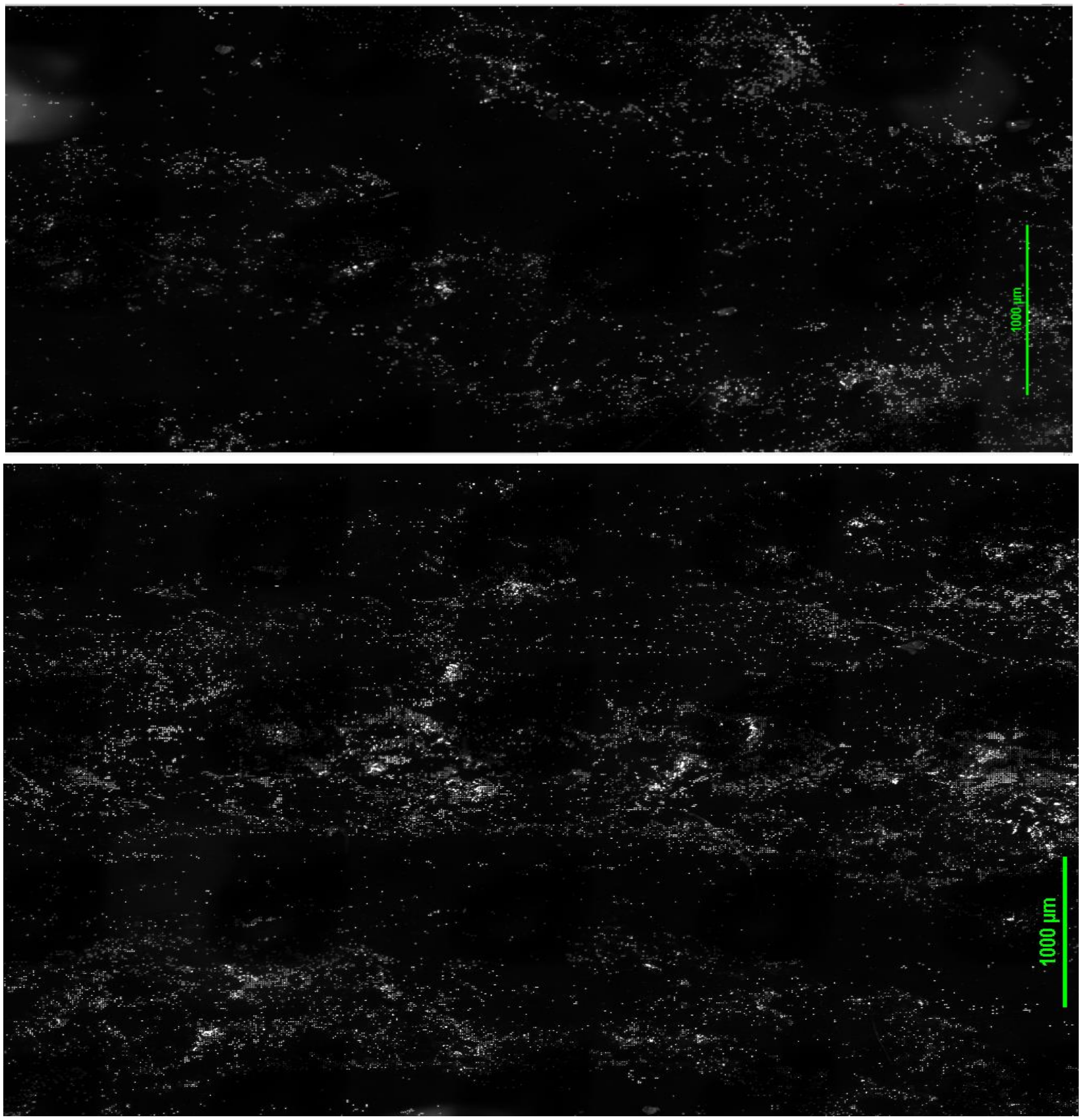

Figure S4. Composite fluorescence micrographs showing failure to contain microsphere solution of channels produced from 3D printed moulds,

CAD Designs

Homebrew method:

- Spiral:
- Straight rectangular:

SLA 3D print files:

- Spiral
- Straight rectangular

3D files & all other data available upon reasonable request to the corresponding Author
